# Ion Migration and Dopant Effects in the Gamma-CsPbI_3_ Perovskite Photovoltaic Material: Atomistic Insights through *Ab Initio* and Machine Learning Methods

**DOI:** 10.1021/acs.chemmater.5c00503

**Published:** 2025-06-10

**Authors:** Allison Nicole Arber, Vikram, Felix C. Mocanu, M. Saiful Islam

**Affiliations:** Department of Materials, 1555 University of Oxford, Oxford, OX1 3PH, U.K.

## Abstract

Inorganic halide perovskites such as CsPbI_3_ are attracting increasing attention for solar cell and optoelectronic applications. Ion migration is known to be an important factor in perovskite behavior, but the impact of cation dopants on iodide diffusion in the room-temperature orthorhombic γ-CsPbI_3_ is not fully understood, especially at the atomic level. Here, we investigate the effect on iodide migration of incorporating different cations (including Sn^2+^, Ba^2+^, and Cu^2+^) into γ-CsPbI_3_, focusing on maintaining an inorganic phase rather than doping with molecular organic ions. Through a combination of *ab initio* and machine learning (ML) techniques, our results show that the simulated structure, band gap, and ion migration energies are in good agreement with experimental data. We find that partial Pb-site substitution does not have a major suppressing effect on iodide ion transport, which is important for guiding future doping work. An ML interatomic potential model was derived for large-scale simulations (∼80 ns) of the pristine and Sn-doped materials, which reveal iodide diffusion paths along the Pb–I octahedral edges with no correlated cation motion. Structural analysis indicates an ordered cation sublattice but disorder in the anion sublattice, indicative of high iodide ion mobility similar to fast-ion conductors.

## Graphical abstract

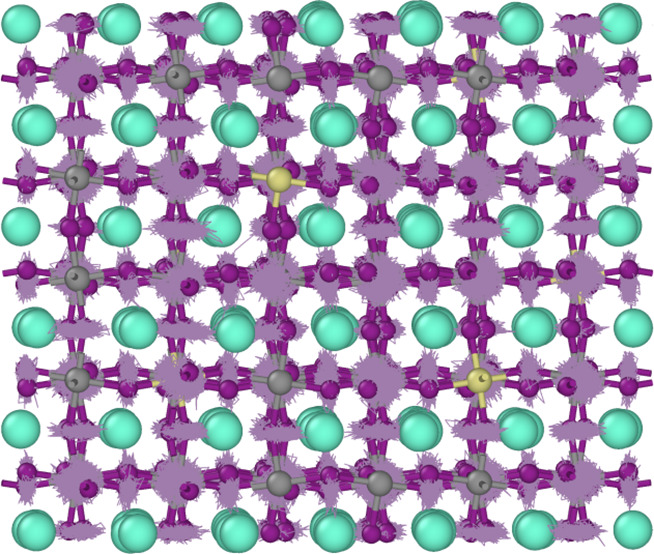

## 1 Introduction

Halide perovskites are prime candidates for next-generation photovoltaic materials, largely due to their record-breaking power conversion efficiencies (PCE).^1,2^ Currently, hybrid organic–inorganic perovskite PCEs have surpassed 26%,^3–5^ while the inorganic perovskite CsPbI_3_ has reached a record of 21.15%.^6,7^ Perovskite solar cells, in contrast to silicon, benefit from easier and more cost-effective fabrication via either solution processing or vapor deposition.^8–11^ The tunability of perovskite band gaps by elemental substitution also makes them excellent materials for use in single as well as multijunction solar cells.^12,13^

In particular, inorganic halide perovskites have higher thermal stability and are more resistant to degradation under light and high humidity than their hybrid counterparts.^14,15^ This allows some CsPb*X*_3_ (*X* = Cl, Br, I) compositions fabricated under atmospheric conditions to demonstrate 1–2 months of stability with minimal loss of efficiency^14,16^ even without encapsulation.^14^ This higher stability has sparked interest in γ-CsPbI_3_ for single-junction cells. In addition, the band gap of 1.73 eV^17,18^ is ideal for both silicon-perovskite and perovskite–perovskite tandem solar cells to push PCEs above the theoretical limits.^19,20^ Inorganic lead halide perovskites have also gained interest for their applications in quantum dots and light-emitting devices due to their narrow emission and bright photoluminescence.^21–26^

Despite the increased stability of inorganic perovskites, these materials suffer from high intrinsic point defect concentrations (∼10^14^ cm^–3^)^27,28^ which are dominated by halide vacancies. Halide vacancy-mediated diffusion has been shown to be the dominant mechanism for long-range ion transport in these perovskite systems.^29–32^ This has been supported by the low activation energies (∼0.3 eV) for halide migration, as demonstrated by ionic conductivity studies^29–31^ on similar inorganic perovskite halides (e.g., CuPbI_3_ and CsPbBr_3_). Intrinsic halide ion migration is known to be a leading cause of structural degradation^33^ and voltage–current hysteresis,^33–37^ both of which lead to a decrease in PCE over the cell’s lifetime.

Recent studies^38–58^ have found that certain cation dopants can modify structural stability and device performance, but there has not been any comprehensive study comparing the atomic-scale effects of cation dopants on ion migration barriers in CsPbI_3_. The presence of dopants can introduce strain into the lattice, which can affect the iodide migration pathways and their activation energies. Inorganic dopants are of particular interest, as they can create such effects without introducing the volatility concerns associated with molecular organic cations.^13,14^

Previously related computational work on CsPbI_3_ ^59,60^ has largely focused on the cubic α-phase^61–64^ which exists above 550 K,^65^ with limited modeling studies on other phases.^41,66–69^ However, structural studies^17,65,70^ have demonstrated that, at room temperature, CsPbI_3_ adopts the orthorhombic γ-polymorph perovskite structure (Figure 1a). The orthorhombic symmetry allows different possible migration pathways, as the iodide ions occupy two inequivalent Wyckoff positions with symmetries 4c and 8d. As such, there is a need for a comprehensive study that extends our understanding of ion migration to the γ-phase of the inorganic perovskite and explores the effects of dopants.

**Figure 1:**
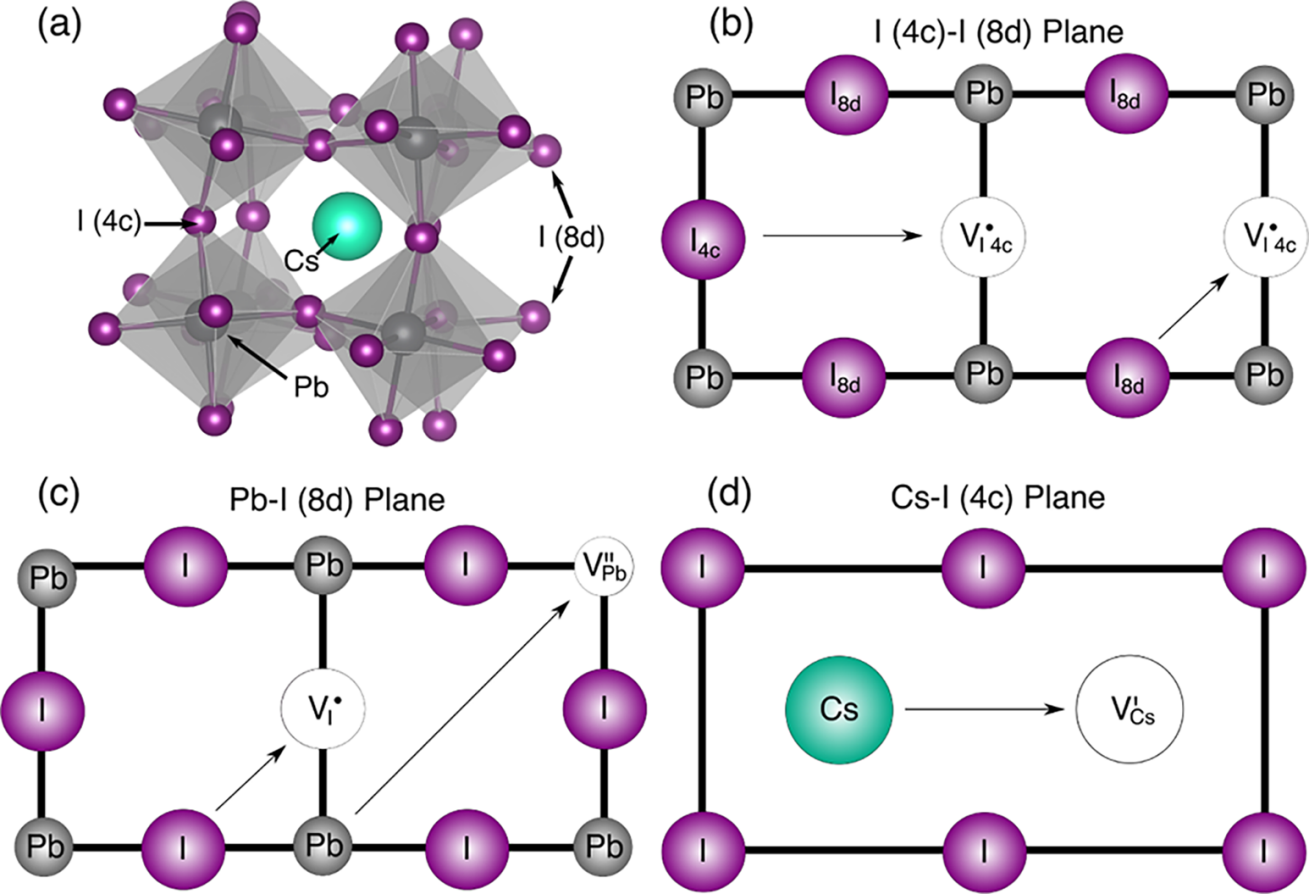
(a) Structure of γ-CsPbI_3_ contains cesium cations, coordinated by 12 iodide ions, which are held within a lattice of PbI_6_ octahedra. (b–d) The five migration pathways investigated in this work including I–I pathways between 8d and 4c sites.

By focusing on the room-temperature orthorhombic phase (γ-CsPbI_3_), this work investigates the various pathways of ion transport in the pristine material and explores the effects of a range of cation dopants using advanced atomistic modeling techniques. We first used *ab initio* density functional theory (DFT) calculations to model the structure of the pristine and doped γ-CsPbI_3_ perovskite including the presence of iodide defects. Activation energy barriers associated with various iodide migration pathways were then obtained for a range of cation dopants, such as Sn^2+^, Cu^2+^, and Ba^2+^. Although our focus is on isovalent 2+ cation substitution, certain aliovalent dopants (Ag^+^, Bi^3+^, Sb^3+^) have been investigated experimentally^39,45,71^ and are included in our modeling analysis for direct comparison.

To investigate ion diffusion over very long time scales (∼80 ns) that are inaccessible using *ab initio*-based techniques, molecular dynamics (MD) simulations were performed for the first time on pristine and Sn-doped γ-CsPbI_3_ using machine-learned interatomic potentials (MLIPs) trained with MACE,^72^ a message-passing neural network (as detailed in the Methods section).

## 2 Results and Discussion

### 2.1 Ion Transport in Pristine γ-CsPbI_3_

Developing an accurate simulation model of the crystal structure and electronic band gap was the starting point of this study. Our model for γ-CsPbI_3_ shows good agreement with experimental data, reproducing the structural lattice parameters^17^ to within 2% and the band gap to within 0.13 eV of the measured value from photoluminescence studies^13^ (Table 1). We then investigated the formation energetics of the full Schottky and iodide Frenkel defects. The Schottky defect consists of a formula unit of vacancies, and the Frenkel defect is formed by a vacancy and interstitial pair (eqs. S1, S2). We found the CsPbI_3_ Schottky defect to have the lowest energy (0.18 eV/defect) and hence to be more stable compared to the iodide Frenkel (0.58 eV/defect), in good agreement with previous work on perovskite iodides.^73^ These values support the current understanding that Schottky-type defects lead to the high intrinsic defect densities in these perovskites, causing the vacancy-mediated diffusion of iodide ions.^29–32^ It is worth noting that long-range iodide interstitial diffusion in inorganic perovskite halides (or oxides) has not been observed experimentally due to the lack of interstitial space for large anions in such close-packed structures. Indeed, iodide vacancies as the primary mobile point defects were confirmed by measurements of the ionic conductivity as a function of iodine partial pressure.^32^

**Table 1:** Comparison of Experimental^13,17^ and Calculated Structural Properties (Lattice Constants, Angles, and Bond Distances) and Band Gap of γ-CsPbI_3_

Parameter	Experiment	Calculated	Difference
*a*/Å	8.86	8.71	–0.15
*b*/Å	8.58	8.68	0.10
*c*/Å	12.47	12.44	–0.03
Mean angle/°	90.00	90.00	0.00
Cs–I bond/Å	4.42	4.43	0.01
Pb–I bond/Å	3.18	3.18	0.00
Band gap/eV	1.72	1.60	0.12

As noted, the symmetry of the room-temperature stable orthorhombic γ-CsPbI_3_ lattice allows for different possible migration pathways, which have not been previously studied. Figure 1 illustrates the various Wyckoff symmetry sites in the γ-CsPbI_3_ lattice (Figure 1a) and the ion migration pathways investigated in this work, which involve conventional hopping between neighboring lattice sites (Figure 1b–d).^33^ The pathways investigated were (i) I migration between neighboring sites in the Cs–I plane (4c site); (ii) I migration along an octahedron edge between the Pb–I plane (8d site) and the Cs–I plane (4c site); (iii) I migration along an octahedron edge in the Pb–I plane (8d site); (iv) Pb migration along the diagonal <220> direction; and (v) Cs migration into a neighboring A-site vacancy within the Cs–I plane (4c site). The plane comprised of Pb and I 8d atoms (Figure 1c) is parallel to the Cs–I plane (Figure 1d) and contains the migration pathways of both Pb and I 8d ions to their respective vacancy sites. The plane containing both symmetry iodide sites (Figure 1b) is orthogonal to the other two planes and shows the migration of I 4c ions as well as transport between the two iodide sites. Energy barriers can be derived for these mechanisms by performing a series of transition-state calculations between adjacent sites. The calculated activation energies for these migration pathways are listed in Figure 2.

**Figure 2:**
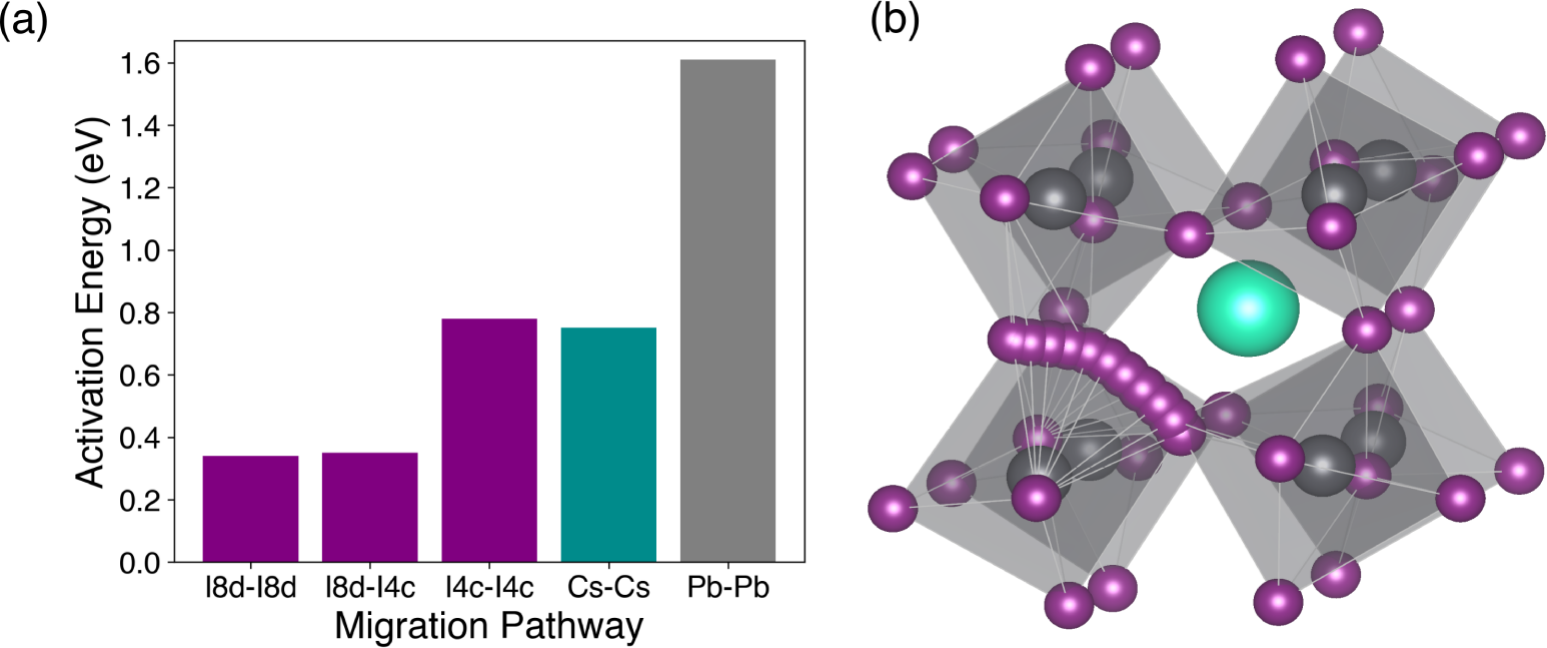
(a) Activation energy barriers for all the possible migration pathways (illustrated in Figure 1) in γ-CsPbI_3_; (b) the I8d-I8d migration pathway; the most favorable pathways correspond to I migration between 8d-8d or 8d-4c sites, following a slightly curved path.

We found that iodide ion migration along the PbI_6_ octahedral edge has the lowest activation energies of 0.34 and 0.35 eV between 8d-8d and 8d-4c sites, respectively. Energy profiles for the I migrations are shown in Figure S1. The comparatively high activation barriers for Cs and Pb indicate that these migrations are less likely, suggesting an immobile cation sublattice. We also found that the long I 4c-4c migration path has a high activation energy, which strengthens our understanding that I migration occurs only along the PbI_6_ octahedral edges. These energies correlate well with values from previous experimental and computational studies of inorganic halide perovskites summarized in Table 2 with most values falling in the range of 0.3–0.4 eV for Pb-based perovskites. These results confirm that halide ions are the dominant migrating species in these perovskites, with relatively low migration barriers. Interestingly, analysis of the migration paths in γ-CsPbI_3_ reveals a small deviation from the linear route, involving a curved path between iodine sites with the saddle point slightly bowed away from the adjacent Pb ion (shown in Figure 2). This atomic-scale feature is difficult to observe using experimental techniques alone. Such curved migration paths have been found previously in modeling studies of MAPbI_3_^33^ and the LaGaO_3_ perovskite oxide-ion conductor,^74^ which were subsequently confirmed by experimental neutron diffraction maximum entropy methods.^75^

**Table 2:** Summary of Experimental and Calculated Activation Energies for Halide Ion Migration in a Range of Inorganic Halide Perovskites from Previous Studies and This Work

Compound	Activation Energy (eV)	Technique/Reference
CsPbCl_3_	0.29	Expt: ionic conductivity^29^
CsPbBr_3_	0.25	Expt: ionic conductivity^29^
KMnCl_3_	0.39	Expt: ionic conductivity^29^
CuCdCl_3_	0.36	Expt: ionic conductivity^76^
CsPbCl_3_	0.17	Expt: ionic conductivity^30^
CsPbBr_3_	0.17	Expt: ionic conductivity^30^
KMnCl_3_	0.65	Expt: ionic conductivity^77^
CsCdCl_3_	0.73	Expt: ionic conductivity^77^
CsSnBr_3_	0.23	Expt: ionic conductivity^77^
CuPbI_3_	0.29	Expt: ionic conductivity^31^
CsPbI_3_	0.38	Expt: selective blocking^78^
α-CsPbI_3_	0.36	Calc: DFT^37^
α-CsPbBr_3_	0.44	Calc: MD^79^
α-CsPbBr_2_I	0.33	Calc: MD^79^
α-CsPbBrI_2_	0.49	Calc: MD^79^
α-CsPbI_3_	0.53	Calc: MD^79^
γ-CsPbI_3_	0.34	Calc: DFT-NEB (this work)
γ-CsPbI_3_	0.42	Calc: MLIP (this work)

### 2.2 Effect of Dopants on Ion Migration Barriers

Benchmarking the migration pathways in the pristine structure allowed us to explore the effect of various dopants on the iodide migration pathways and the activation barriers. Previous experimental studies^38–58^ propose a variety of inorganic dopants to improve photovoltaic (PV) performance, but the precise effects on ion migration have not been fully characterized. Here, we extend our related modeling work on ion migration in pristine and doped MAPbI_3_ and FAPbI_3_ perovskites^33,38,80–82^ to provide a comprehensive comparison of the effect of cation dopants on I migration energies in γ-CsPbI_3_. A range of B-site dopants were investigated, including Ba^2+^, Cu^2+^, Zn^2+^, Ge^2+^, Cd^2+^, and Sn^2+^ at a typical low 3% concentration to retain the same 3D perovskite structure and because phase segregation can occur at high dopant substitution levels. The pathway studied in each case was the I vacancy migration between adjacent 8d symmetry sites, which we found has the lowest activation energy for migration and allowed us to probe trends in ion migration energies in a systematic manner. These B-site dopants were placed at the center of the octahedron within which the migration occurs.

The resulting ion migration activation barriers (Figure 3) indicate three main features. First, none of the wide range of cation dopants led to an appreciable increase in activation energy, and in fact, several dopants decreased the activation energy. Hence, the results suggest that dopants such as Zn would not significantly suppress iodide ion migration in γ-CsPbI_3_, which is important in guiding future doping studies. Second, structural analysis at the atomistic level found that dopants with a high barrier such as Cd or Sn have rigid structures during ion migration, whereas dopants like Zn cause local structural relaxations, resulting in lower energy iodide ion migration. Interestingly, the dopant-iodide bond distances at the transition state around the Zn dopant are reduced by ∼0.3 Å in comparison to those of Cd and Sn. Other structural properties were also analyzed, including the dopant ion radius and the dopant-iodide bond length at the migration transition state. There do not appear to be any overarching design rules that predict the trends in activation barriers for these dopants, although the magnitude of the energy differences is relatively small. A key feature that differentiates the highest-barrier pathways from the lowest barrier pathways is the lack of any structural rearrangement before or after iodide ion migration. Third, the dopants Cd and Sn cause small increases in the activation energy when doped on the Pb site. While Cd results in a higher activation energy, its high toxicity^83^ makes it an undesirable dopant. In contrast, Sn is a promising candidate due to the increasing interest of the scientific community in mixed Pb–Sn perovskites.^84^ Mixing Sn in suitable quantities (∼50–60%) at the Pb site improves carrier mobility and allows stronger optical absorption by reducing the band gap. The band gap tunability allows mixed Pb–Sn perovskites to be used in stand-alone or tandem solar cell applications. Although beyond the scope of the current investigation, it is worth noting that there has been recent interest in the role of oxidized tin (Sn^4+^) in suppressing ion migration^82^ as well as the use of molecular additives or defect passivation to improve the efficiency and stability of CsPbI_3_ solar cells.^6,15,85^

**Figure 3:**
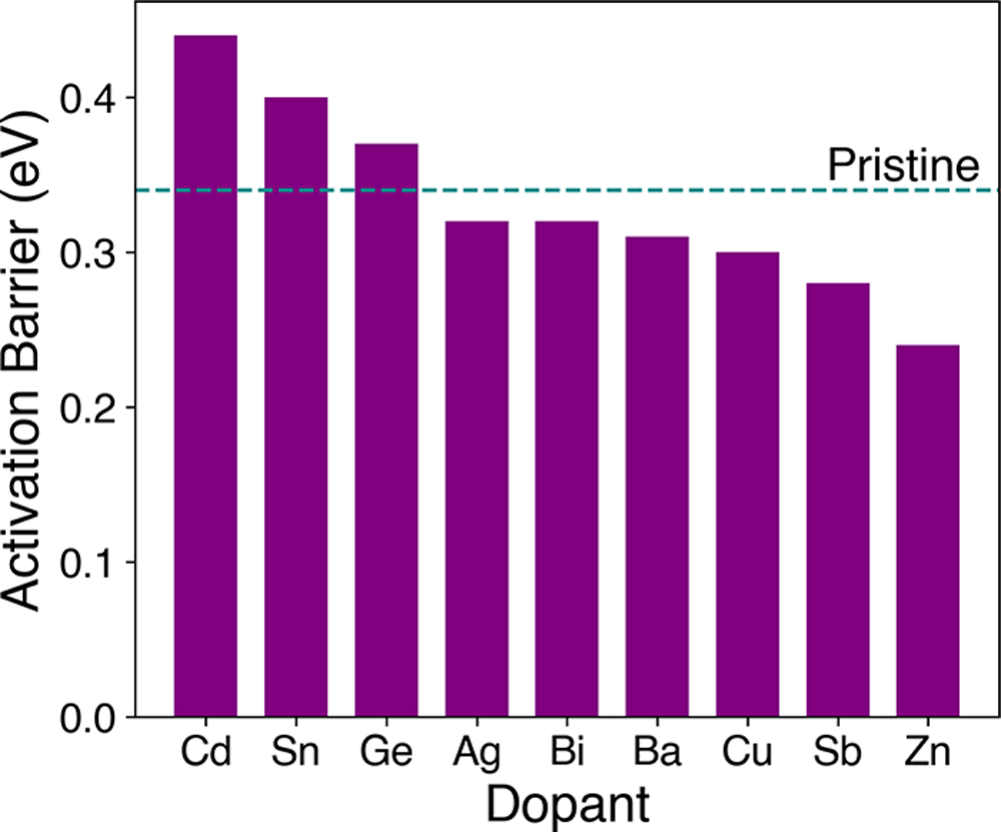
Activation energies for I vacancy migration in γ-CsPbI_3_ between neighboring 8d sites near a dopant atom at the Pb site.

### 2.3 Machine Learning Model of Ion Diffusion in γ-CsPbI_3_

An important factor for understanding the transport flux through the perovskite absorber layer is the rate of iodide ion diffusion. To complement the migration barrier simulations and to gain insights into long-range diffusion rates, we employed a machine learning (ML) architecture of message-passing atomic cluster expansion (MACE)^72^ to derive a single ML interatomic potential (MLIP) model (detailed in Methods). The model was used to run MD simulations of pristine and 3% Sn-doped γ-CsPbI_3_. As noted, we focus here on the Sn dopant due to the growing interest in mixed Pb–Sn perovskites for band gap tunability and for potential application in tandem solar cells.

Using the trained MLIP, long-time-scale MD simulations were performed on pristine and Sn-doped γ-CsPbI_3_ compositions, which have not been widely applied to examine ion diffusion in inorganic perovskite halides. Each of these compositions included representative concentrations of iodide vacancies, in line with the DFT calculations (Figure S2). While maintaining quantum mechanical accuracy at reduced computational cost, the MD simulations were run for a temperature range of 300–500 K and for a long time scale of ∼80 ns, which is orders of magnitude greater than current *ab initio* MD. Diffusion coefficients (*D*_I_) for the mobile iodide ions were derived from the temperature-dependent mean square displacements (MSDs) (Figure S3) and the activation barriers were calculated via the Arrhenius relationship, as displayed in Figure 4. MSD analysis demonstrates that ∼80 ns is sufficient to achieve the conversion (Figure S4).

**Figure 4:**
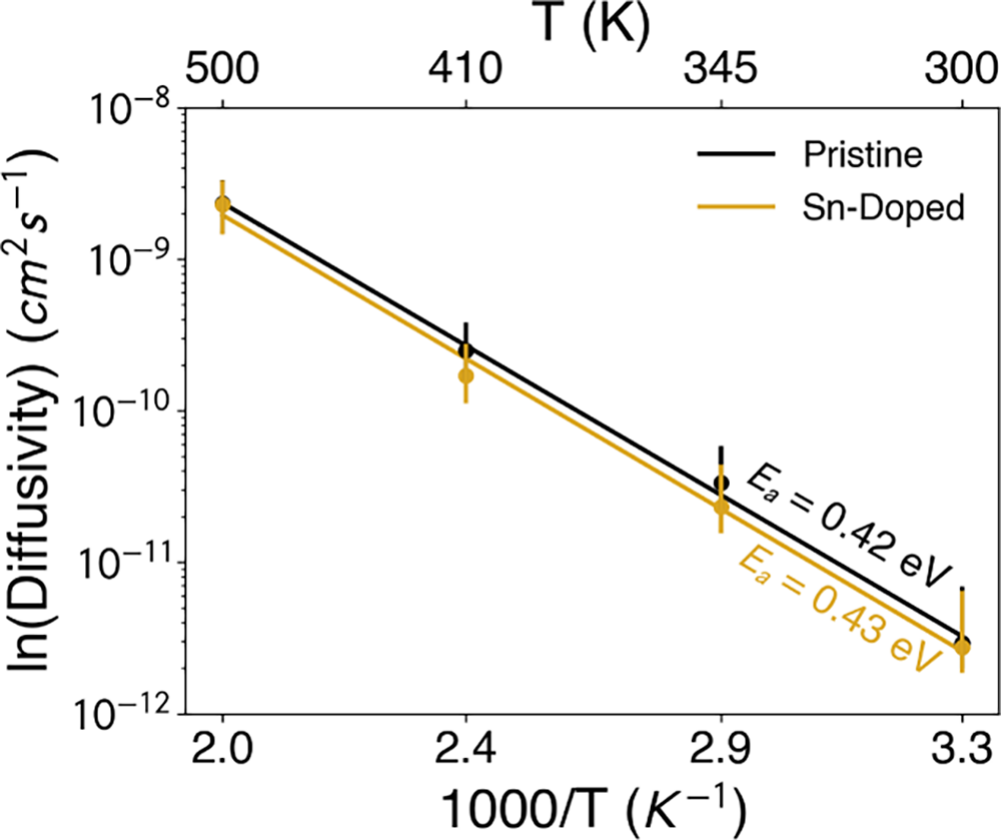
Arrhenius plots of the calculated diffusion coefficients for pristine and 3% Sn-doped γ-CsPbI_3_.

The resulting activation barriers of 0.42 and 0.43 eV for the pristine and Sn-doped systems, respectively, are in good agreement with experimental values (Table 2) and with our DFT results. We note that, despite the small difference in activation energies from MD vs DFT-NEB, these values are still within the range of energy barriers experimentally found in similar inorganic perovskite systems and show consistent trends with temperature and composition. In DFT-NEB calculations, the migration barriers are computed along a predefined path, which means migrations around Sn are explicitly considered. MD simulations are more representative of experimental conditions, where ion migration occurs via multiple pathways. Given 3% Sn-doping, most of the halide migration still occurs around Pb sites, leading to an average migration barrier that remains similar to the pristine system. We note that, even though Sn-doping can improve efficiencies by reducing the band gap, the similar iodide migration barriers for the pristine and Sn-doped material imply that small amounts of Sn-doping would not directly resolve the long-term stability issue of ion migration-related degradation. Direct comparison with experimental D_I_ values can be difficult, as they are not straightforward to measure for complex perovskites. Indeed, experimental D_I_ values for the perovskite MAPbI_3_ range between 10^–11^ and 10^–6^ cm^2^s^–1^ at room temperature^86^ which may reflect differences in experimental conditions, thermal history, and phase purity.

Further atomistic insights into the mechanistic features of iodide-ion transport can be gained from visualizing the ion jumps or trajectories from the MD simulations (shown in Figure 5). This illustrates the significant movement of iodide ions in the pristine and Sn-doped structures. The line trajectories also confirm that iodide-ion diffusion occurs via conventional hops between iodide sites along the Pb–I octahedral edges for both systems, in agreement with our DFT simulations. The iodide mobility is not accompanied by any distinct correlated motion of the Pb or Cs ions.

**Figure 5:**
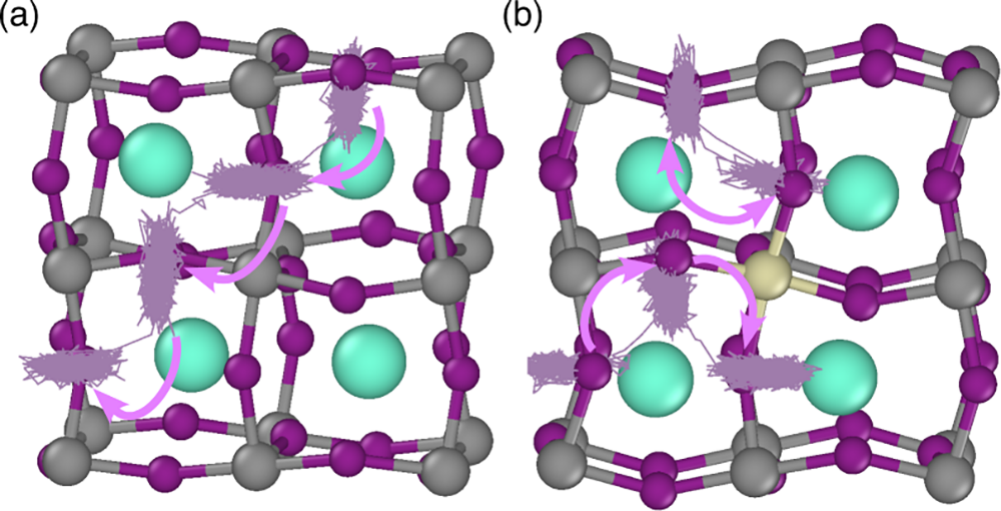
Iodide ion trajectories from the MD simulations (with 1% iodide vacancies) superimposed on the relaxed γ-CsPbI_3_ lattice to illustrate mechanistic features through (a) the pristine lattice and (b) the Sn-doped lattice.

Additional structural information can be gleaned from the radial distribution function (RDF), which provides insights into the long-range order (or disorder) of perovskite materials. The RDFs for Pb–Pb (shown in Figure 6) reveal a series of sharp peaks for both compositions, with the first coordination shells clearly resolved, in agreement with the observed ordered crystal structure of γ-CsPbI_3_. In contrast, the RDF for I–I shows a broader, more diffuse structure for separations greater than nearest-neighbor, indicative of greater ion mobility in the iodide sublattice. Additional analysis of the visualized trajectories for all elements, as well as the elemental MSDs, confirms that iodide mobility dominates (Figures S5 and S6). This is more pronounced at higher temperatures, showing lower maxima (Figure 6c). Interestingly, such features are similar to those found in fast-ion-conducting solid electrolytes for fuel cells and lithium batteries, in which the RDFs of the mobile sublattice are commonly more disordered than those of normal crystalline solids.

**Figure 6:**
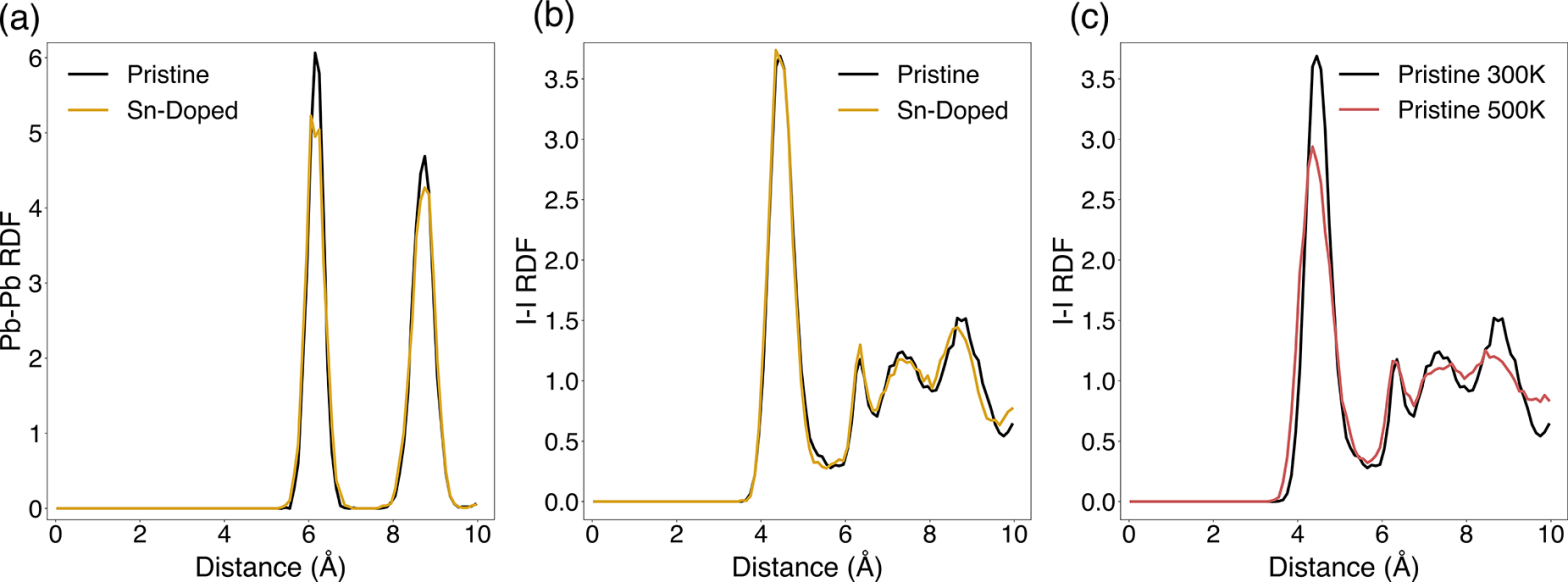
Radial distribution functions (RDFs) of (a) Pb–Pb and (b) I–I in the pristine and Sn-doped compositions at 300 K. (c) I–I (RDFs) for the pristine composition at 300 and 500 K.

## 3 Conclusion

Deeper atomic-scale understanding of doping and transport phenomena in inorganic perovskite halides is important for the development of their optoelectronic applications. In this study, we investigated the effect on iodide ion migration of incorporating different cation dopants into orthorhombic γ-CsPbI_3_.

Three key conclusions emerge. First, the orthorhombic structure, band gap, and ion migration energy from the *ab initio* simulations of γ-CsPbI_3_ are in good agreement with the available experimental data. The lowest migration activation energy was observed for the iodide ion vacancy pathway between adjacent 8d symmetry sites. Second, by investigating trends in a systematic manner, we find that none of the partial B-site dopant substitutions (including Sn^2+^, Ba^2+^ and Cu^2+^) give rise to an appreciable increase in activation energy for iodide migration; certain dopants such as Zn^2+^ lead to lower migration barriers due to local structural relaxations. The results, therefore, suggest that these dopants would not significantly suppress iodide ion migration in γ-CsPbI_3_. Third, a state-of-the-art machine learning interatomic potential model was derived for large-scale MD simulations (∼80 ns) of the pristine and Sn-doped materials, which have not been widely employed to examine diffusion mechanisms in inorganic perovskite halides. The results revealed iodide diffusion paths along the Pb–I octahedral edges with no evidence of correlated Pb or Cs motion. Such mechanistic features are difficult to extract from experiments alone. Structural analysis indicates long-range order of the Pb sublattice in agreement with the observed crystalline structure and disorder in the anion sublattice, indicative of high iodide ion mobility. Overall, this study enhances our fundamental understanding of γ-CsPbI_3_ and provides a framework to guide future doping and ion transport work on these inorganic perovskite solar cell materials to improve their device performance.

## 4 Methods

### 4.1 *Ab Initio* Methods

DFT calculations were performed using the Vienna Ab initio Simulation Package (VASP),^87–90^ a plane-wave code with projector-augmented-wave (PAW) pseudopotentials^91,92^ which has been extensively used for modeling perovskite solid-state materials. The generalized gradient approximation (GGA) exchange-correlation functional PBEsol^93^ was used for all DFT calculations, with a plane-wave cutoff energy of 500 eV and a k-point mesh corresponding to a k-spacing of 0.2/2π Å^‑1^. Forces were converged to less than 0.01 eV/Å for all structural optimizations. PBEsol was chosen because it provided a balance of computational efficiency and accuracy in replicating structural parameters compared to X-ray diffraction experiments (Table 1).^17^ All calculations included spin polarization to ensure that any effects due to unpaired electrons, notably in the defect structures, were captured.^94^ Functionals at higher levels of theory were also tested but did not improve the accuracy of predicting structural parameters.

The ion migration pathways were modeled using climbing image nudged elastic band (CI-NEB) calculations^95,96^ in VASP. These calculations were performed on 2 × 2 × 2 supercells containing 159 atoms (due to the inclusion of a single I vacancy) which ensured minimal interactions between repeating images. The activation energy of a migration pathway was calculated by subtracting the starting point energy from the energy at the position of highest potential energy, the “saddle point.” In each case, the starting and end points were well-converged to avoid inaccuracies in the transition state energy profile. Due to a sufficiently large supercell, these calculations represent migration in the dilute limit without any interactions between migrating ions. This approach has been used successfully in numerous ion migration studies.^33,38,81^

### 4.2 Machine Learning Methods

The machine-learned interatomic potentials (MLIPs) were trained on ∼4,000 structures (Table S1) taken from *ab initio* molecular dynamics (AIMD) simulations and single points derived from the CI-NEB calculations. The energies and forces of each structure were calculated at the same GGA level used for previous DFT calculations. The structures included in the training set covered the pristine system as well as defect configurations containing vacancies and dopants. These MLIPs were trained using MACE, a state-of-the-art equivariant graph neural network model.^72,97^ The model has a maximum equivariance order of two, 256 chemical channels, and two layers with a cutoff radius of 5 Å. A single potential model was trained from scratch, which is transferable between the compositions of interest. Evaluations of the model training are shown in Figure S7.

### 4,3 Diffusion Analysis

The MD simulations were run in LAMMPS^98^ on supercells containing 960 atoms with 1% iodide defects at constant volume and constant temperature for ∼80 ns. We stress that this simulation timescale is much longer than standard *ab initio* MD simulations, which are typically less than 100 ps. Ionic diffusivities for the iodide ions were extracted from trajectories at four different temperatures arranged linearly on an inverse temperature scale between 300 and 500 K. The MSD plots (Figure S3) and diffusion coefficients were calculated using Kinisi version 1.0.0.^99,100^ The diffusion coefficients were calculated from the MSD plots using the following relationship:

1 $$\mathrm{MSD}={\langle \left(x\left(t\right)-x\left(0\right)\right)}^{2}\rangle =6Dt$$

and the Arrhenius relationship (eqs. S1, S2) was used to calculate the activation barrier from the diffusion coefficients. Using the form given below, the activation barrier can be found from the slope of the natural log of the diffusion coefficients when plotted vs 1/T.

2 $$\ln D=-\frac{E_{a}}{RT}+\ln D_{0}$$

## Supplementary Material



## Notes

Schottky and Frenkel defect equations, DFT-NEB energy profiles, and additional MLIP information (structures, MSD plots, and training data)­(PDF)
