# Supplementary material for: Ion Migration and Dopant Effects in the Gamma-CsPbI3 Perovskite Photovoltaic Material: Atomistic Insights through Ab Initio and Machine Learning Methods
Source: Chem Mater. 2025 Jun 10;37(12):4416–24. doi: 10.1021/acs.chemmater.5c00503 (PMC12199300; doi:10.1021/acs.chemmater.5c00503)
Supplement: Supplementary file 1 [file cm5c00503_si_001.pdf]

Supporting Information

Ion Migration and Dopant Effects in the Gamma-$CsPbI_3$ Perovskite Photovoltaic Material: Atomistic Insights through Ab Initio and Machine Learning Methods

*Allison Nicole Arber, Vikram, Felix C. Mocanu, M. Saiful Islam**

Department of Materials, University of Oxford, Oxford, OX1 3PH, United Kingdom

Equations S1 and **S2** represent the formation of the Schottky and Frenkel defects, respectively.

Schottky: $$nil \rightarrow V_{Cs}' + V_{Pb}'' + 3V_I^{\cdot} + CsPbI_3$$ Eqn. S1

I Frenkel: $$nil \rightarrow V_I^{\cdot} + I_I'$$ Eqn. S2

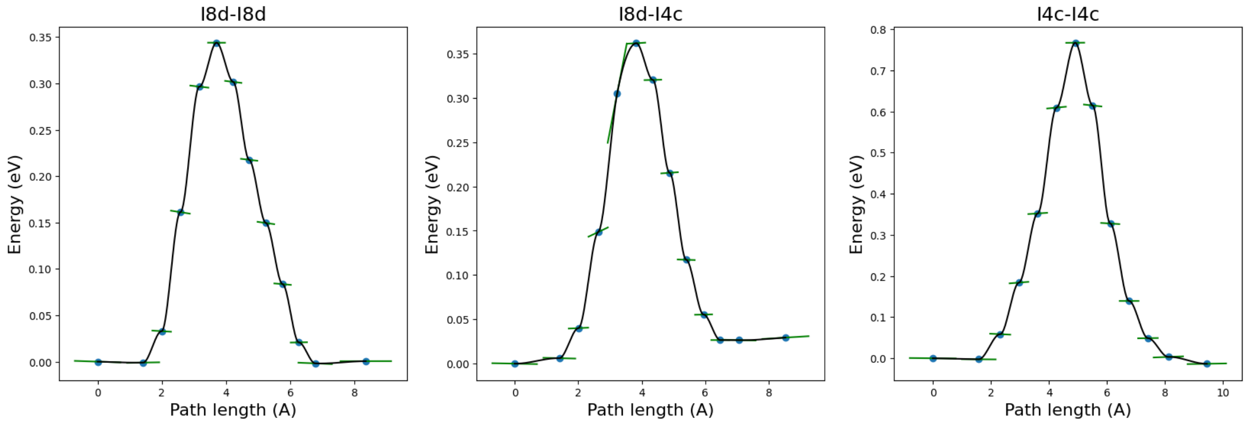

Figure S1. Energy profiles for the three iodide migration pathways investigated in the pristine compositions. The average of the forward and backward barrier is the reported value for all activation energies given in the text. For these three pathways, the 8d-4c transition is the only one with slightly different forward and reverse barriers; although, the difference is only about 0.03 eV.

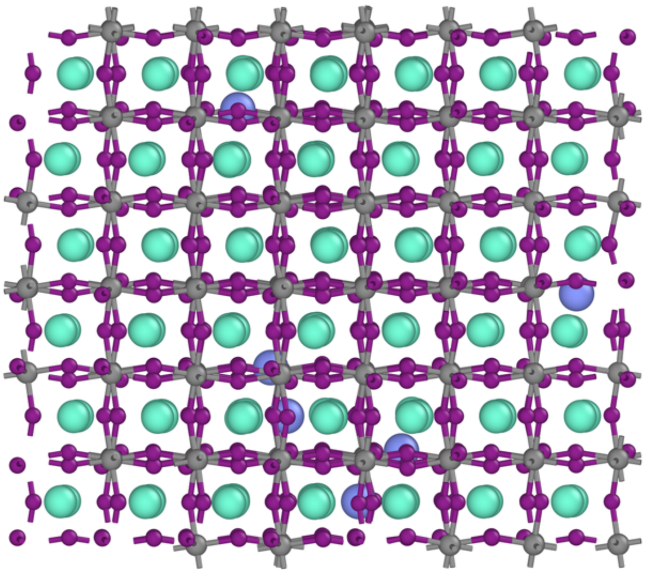

Figure S2. The starting geometry of the MD simulation for the pristine γ-$CsPbI_3$ composition. The sites of the iodide vacancies are marked in blue. These positions were selected randomly to set the vacancy concentration to 1% in the supercell.

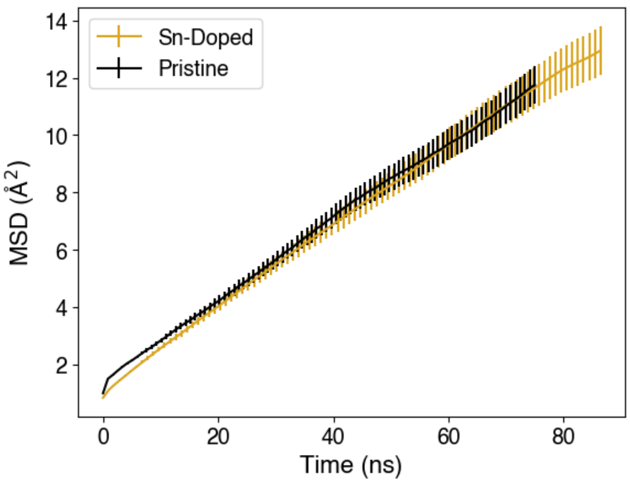

Figure S3. MSD plot showing the ionic diffusion for the pristine and Sn-doped compositions at 500 K.

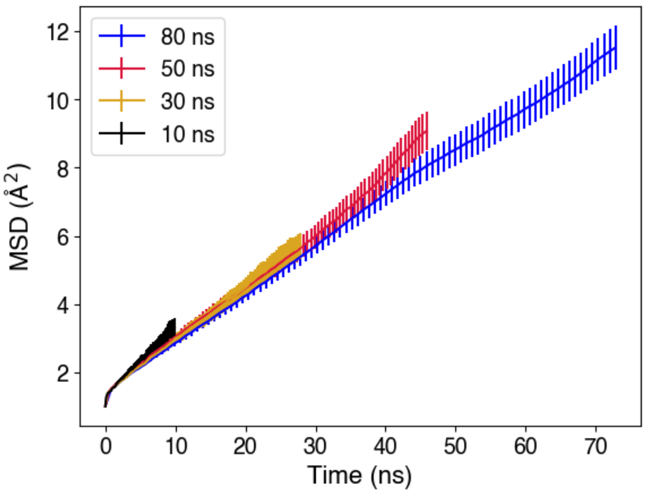

Figure S4. MSD of the 500K pristine molecular dynamics trajectory plotted over 10, 30, 50, and 80 ns demonstrating conversion over the simulation time.

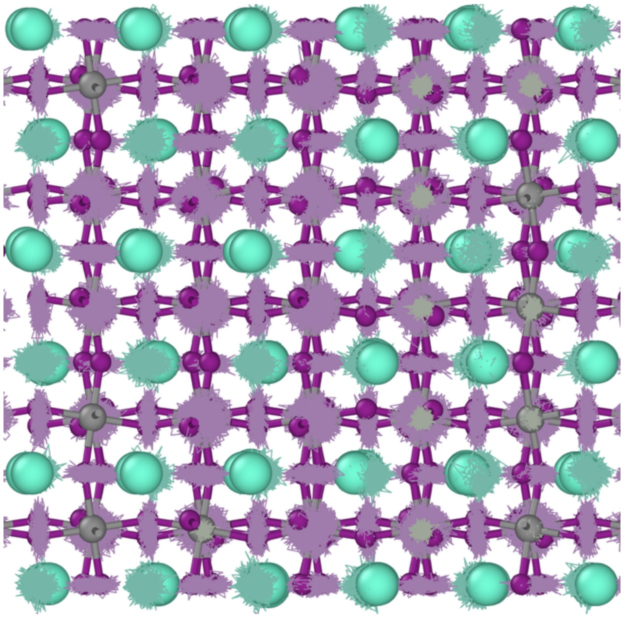

Figure S5. Trajectories of all species over a time period of 0.1 ns. In agreement with the calculated RDFs (**Figure 7**), the motion of the iodides (light purple) dominates the system with the cesium (aqua) and lead (gray) atoms displaying a smaller degree of motion around their lattice sites.

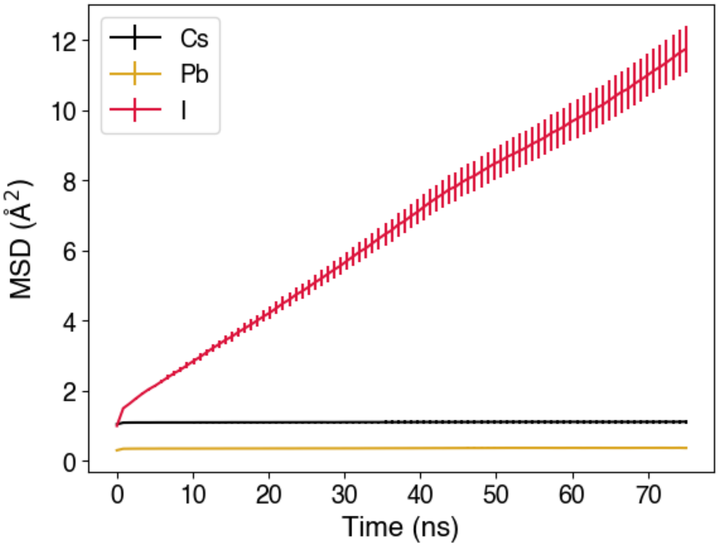

Figure S6. Comparison of the elemental MSDs calculated for Cs, Pb, and I from the same 500K trajectory which demonstrates the lack of Cs and Pb movement within the lattice.

Table S1. Number and description of the structures comprising the training dataset according to composition and structure generation source. All structures included are 2x2x2 supercells of the γ-$CsPbI_3$ unit cell. AIMD and MD structures were collected at a range of temperatures between 300-600K.

Pristine				Sn-Doped		
NEB	AIMD		MD	NEB	AIMD	MD
238	NPT 2375	NVT 1274	361	138	NPT 213	90

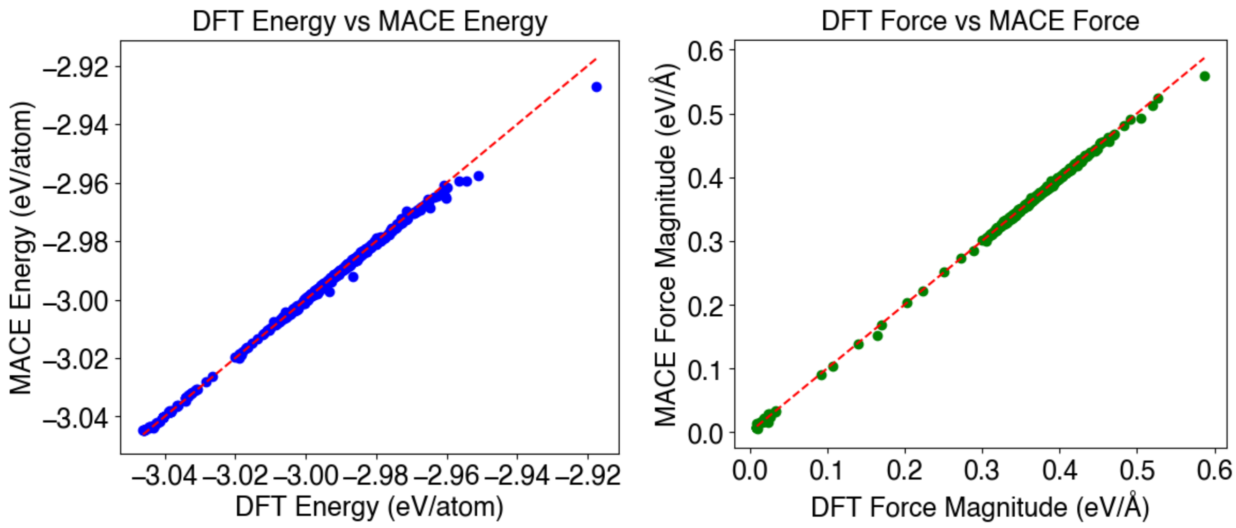

Figure S7. Evaluations of the MACE training to derive a ML interatomic potential which show a mean absolute error in energy of 0.503 meV/atom and a mean absolute error in force of 1.06 meV/A.
